# Ganoderic acid a derivative induces apoptosis of cervical cancer cells by inhibiting JNK pathway

**DOI:** 10.1016/j.chmed.2024.07.002

**Published:** 2024-07-19

**Authors:** Mengchen Wang, Qin Han, Xuelian Zhang, Xi Dong, Jiadong Ran, Fei Wei, Yun Luo, Xiaobo Sun

**Affiliations:** a Institute of Medicinal Plant Development, Peking Union Medical College and Chinese Academy of Medical Sciences, Beijing 100193, China; b Key Laboratory of Bioactive Substances and Resources Utilization of Chinese Herbal Medicine, Ministry of Education, Beijing 100193, China; c Beijing Key Laboratory of Innovative Drug Discovery of Traditional Chinese Medicine (Natural Medicine) and Translational Medicine, Beijing 100193, China; d NMPA Key Laboratory for Research and Evaluation of Pharmacovigilance, Beijing 100193, China

**Keywords:** anti-cancer, apoptosis, cervical cancer, ganoderic acid A derivative, JNK pathway

## Abstract

**Objective:**

Ganoderic acid A can inhibit the proliferation and promotes the apoptosis of cancer cells. Surprisingly, the molecular mechanisms underlying the anti-cancer effects of ganoderic acid A still remain poorly defined. Ganoderic acid A derivative (GaAD19) is an effective ingredient obtained by structural modification of ganoderic acid A. The purpose of this study was to evaluate the anti-proliferation effect of GaAD19 on cervical cancer cells.

**Methods:**

Through the HeLa cervical cancer cell model, the drug target of GaAD19 was predicted using the SwissTargetPrediction database and molecular docking. Subsequently, computer analysis results were verified by a series of molecular biology experiments, such as flow cytometry, Western blot, immunocytochemical staining, terminal deoxynucleotidyl transferase-mediated dUTP nick end labeling (TUNEL), quantitative real time polymerase chain reaction (qPCR), and so on. Then, pathway agonists and inhibitors were used to investigate the mechanism of GaAD19. Finally, the mouse model of cervical cancer was established to evaluate the inhibitory effect of GaAD19 on tumor growth in U14 cervical cancer mice.

**Results:**

GaAD19 induced apoptosis and inhibited the growth of tumors. It also blocked the transition from the G_1_ to the S phase of the cell cycle. However, in the presence of a c-Jun *N*-terminal kinase (JNK)agonist, the effects of GaAD19 on the proliferation, apoptosis, and cell cycle transition of cancer cells were suppressed.

**Conclusion:**

This study showed that GaAD19 can play an anti-cervical cancer role by inhibiting the JNK signaling pathway. These results will be helpful in further exploring the mechanism of GaAD19 in the treatment of cervical cancer.

## 1 Introduction

Cancers constitute malignant tumors with abnormal cell differentiation, proliferation, invasion, and metastasis (Ruttanapattanakul et al., 2021). After breast, colorectal, and lung cancers, cervical cancer is the fourth leading cause of cancer-related deaths among women globally (Sung et al., 2021, Chen et al., 2024). Currently, the treatment of cervical cancer is mainly based on the stage of cervical cancer, the patient’s condition, age, fertility requirements, and so on. The common treatment methods for cervical cancer include radiotherapy, chemotherapy, targeted therapy, surgery, and immunotherapy (Bjurberg, Beskow, Kannisto, & Lindahl, 2015). In addition, biomaterials and tumor organoid models also show great potential in tumor therapy (Xiao et al., 2023; Yang et al., 2023). Common therapeutic drugs for cervical cancer include cisplatin, carboplatin, paclitaxel, fluorouracil, topotecan, and so on. However, most of these have certain side effects. For example, certain chemotherapy drugs can cause secondary cancer, induce heart disease, and lead to peripheral nerve damage (Talib et al., 2022), and these drugs may not even have a positive impact on the cancer being treated. This reduces the patient’s quality of life, and thus there is an urgent requirement to find a novel therapy that will not only provide patients with better treatment, but also reduce side effects.

For more than 2 000 years, *Ganoderma lucidum*, a medicinal fungus, has been utilized in China to cure a variety of ailments (Luangharn et al., 2021). The main medicinal components in *G. lucidum* are ganoderma lucidum triterpenes and ganoderma lucidum polysaccharides (Zhang et al., 2023). Ganoderma lucidum triterpenes include ganoderic acid A (GaA), ganoderic acid D, and ganoderic acid F (Liang et al., 2019). GaA is the major medicinal component of ganoderma lucidum triterpenes (Meng et al., 2022, Jia et al., 2023), and also has anti-cancer activity (He et al., 2023). It is widely used in the adjuvant treatment of complex cancers such as lymphoma (Radwan et al., 2015) and prostate cancer (Gill, Kumar, & Navgeet, 2016). However, some studies have shown that the anti-cancer activity of GaA is not strong (Gill et al., 2016, Yang et al., 2018). In order to develop new anticancer drugs, we found that GaA derivatives prepared by structural modification can improve their biological activity. Thus, it may solve the problem of the low bioavailability of GaA, leading to potent anti-cancer activity. At present, there is no report on the molecular mechanism of GaA derivatives exerting anti-cancer effects.

Mitogen-activated protein kinases (MAPKs) are serine/threonine protein kinases that exist widely in cells. The MAPK pathway plays an important role in various cell functions, and it regulates many important physiological effects such as cell growth, differentiation, stress, and metastasis (Qin et al., 2022). The MAPK signaling pathway is closely related to cancer formation (Zhou et al., 2020). Unlike MAPK signaling, the AMPK [adenosine 5′-monophosphate (AMP)-activated protein kinase] signaling pathway can effectively prevent tumor proliferation and metabolism. AMPK is an important kinase that helps maintain the smooth operation of cell physiological activities and regulates energy homeostasis. The PI3K/Akt pathway is also associated with human diseases, including tumors, and regulates cell survival and apoptosis (Toson, Fortes, Roesler, & Andrade, 2022). These protein kinases form a crisscross network that regulate intracellular signaling and the cell cycle. These signaling pathways converge to regulate molecular gene expression and cellular homeostasis (Semba et al., 2020). Importantly, JNKs are involved in a series of important regulatory mechanisms such as cell growth, differentiation, proliferation, and apoptosis, and also participate in cellular stress response. Thus, they play an important role in many physiological and pathological processes (Pua et al., 2022). Recent studies have shown that the dysfunction of the JNK signaling pathway can cause a variety of diseases, such as Alzheimer’s disease (Liu et al., 2020), ischemia reperfusion injury (Cheng, Chiang, Kao, & Huang, 2021), diabetes (Yung & Giacca, 2020) and tumors (Tam & Law, 2021).

In this study, the HeLa cervical cancer cell model combined with a cervical cancer-bearing mouse model was used to investigate the effect of GaA derivative (GaAD19) on the function of cervical cancer cells. Further, we aimed to identify the signaling pathway underlying the impact of GaAD19 on the proliferation of cervical cells, and we specifically probed the role of JNKs.

## 2 Material and methods

### 2.1 Design of GaAD19

We selected GaA as a lead compound for antitumor agent structural optimization. GaA is a typical lanostane-type triterpene with a C-26 carboxylic acid, which can be easily modified to improve the pharmacological activity. Initially, we introduced benzylamine into GaA. To investigate the potency of the more diversified structures and gain further insight into the structure–activity relationship, we also reduced the C-3 ketone group to hydroxyl. In the end, we obtained GaAD19.

#### 2.1.1 Procedure for GaAD19 synthesis-amidation of C-26 carboxylic acid of GaA

GaA (103 mg, 0.2 mmol) was dissolved in 5 mL dichloromethane (DCM). 1-Ethyl-3-(3-dimethylaminopropyl) carbodiimide hydrochloride (EDCI) (48 mg, 0.25 mmol), 1-hydroxybenzotriazole (HOBT) (34 mg, 0.25 mmol), and triethylamine (55 μL, 0.25 mmol) were added successively. After stirring for 5 min at room temperature (RT) (21–25 °C), 3-bromophenethylamine was added and the reaction was stirred for an additional 2 h until completion of the reaction [monitored by thin-layer chromatography (TLC)]. The reaction was concentrated under reduced pressure. Crude product was purified by silica gel column chromatography (DCM: acetone, 6:1; volume percentage) to give acylamide (109 mg, 78%) as white powder.

#### 2.1.2 Procedure for GaAD19 synthesis-reduction of C-3 ketone

The above synthesized amide (100 mg, 0.14 mmol) was dissolved in 2 mL methanol (MeOH) at 0 °C, and sodium borohydride (12 mg, 0.3 mmol) was added in portions. After stirring for 2 h, the mixture was quenched with dilute hydrochloric acid, and concentrated under reduced pressure. Crude product was purified by silica gel column chromatography (DCM: acetone, 4:1) to give GaAD19 (34 mg, 35%) as a colorless oil.

### 2.2 Cell culture

The human cervical cancer HeLa cell line was purchased from Cell Resource Center, Institute of Basic Medical Sciences, Chinese Academy of Medical Sciences (1101HUM-PUMC000011). Human cervical cancer SiHa cell line was purchased from BeNa Culture Collection (BNCC, Beijing, China) (BNCC337881). Mouse cervical cancer U14 cell line was purchased from Meisen Chinese Tissue Culture Collections (MeisenCTCC, Jinhua, China) (CTCC-400–0316). Mouse hippocampal neuronal HT22 cell line was purchased from MeisenCTCC (CTCC-400–0330). Tumor cells were cultured in a cell incubator with 5% CO_2_ at 37 °C (Thermo, Waltham, USA) using Dulbecco’s Modified Eagle’s medium DMEM (Thermo, Waltham, USA) supplemented with 10% fetal bovine serum (FBS) (Gibco, CA, USA) and 1% penicillin–streptomycin (Gibco). After the cells reached 70%–80% confluence, they were subcultured every 1–2 d.

#### 2.2.1 CCK-8 cell proliferation detection

Cells in the logarithmic phase were collected and inoculated into 96-well plates at a density of 10 000 cells/well, 100 μL per well. The cells were incubated at 37 °C and 5% CO_2_ for 24 h. Subsequently, the original culture medium was abandoned, and the GaAD19 and GaA was added into the wells at concentrations of 0, 50, 25, 12.5, 6.25, 3.125, 1.562 5, 0.781 25, and 0.390 625 μmol/L, respectively. Six experiments were repeated for each concentration. The cells were again incubated for 48 h at 37 °C and 5 % CO_2_. Next, Cell counting Kit-8 solution (10 μL) (Sangon Biotech, Shanghai, China) was added to each well and incubated for about 1 h. Subsequently, the absorbance was measured by SYNERGY H1 (Biotek, Vermont, USA) at 450 nm and the cell inhibition rate was calculated.

#### 2.2.2 Colony formation

HeLa cells were cultured in 6-well plates at a density of 1 000 cells/well for 24 h in a 37 °C cell culture chamber with 5% CO_2_. Then they were treated with GaAD19 at different concentrations (0, 5, 10 μmol/L) and cultured in a 37 °C cell incubator with 5% CO_2_ for 5 d. The inhibitor and agonist groups were treated as follows: HeLa cells were cultured in 6-well plates at a density of 800 cells/well for 24 h in a 37 °C cell culture chamber with 5% CO_2_. Then they were treated with 5 μmol/L GaAD19 combined with 60 μmol/L JNK inhibitors (SP600125) or 0.1 μmol/L Anisomycin for 12 h, and then treated with 5 μmol/L GaAD19 for 5 d. When more than 50 cell clones were visible to the experimenter without the aid of any instrument in the 6-well plate, the cell culture was terminated, cells were fixed 12–18 h with 4% paraformaldehyde (Beyotime, Shanghai, China) at 4 °C, and then stained with 0.5% crystal violet (Solarbio, Beijing, China).

#### 2.2.3 Cell cycle analysis

HeLa cells were inoculated into 6-well plates at a density of 1 × 10^6^ cells/well and cultured in a 37 °C cell incubator with 5% CO_2_ for 24 h. After 6 h of treatment with different GaAD19 concentrations (0, 5, 10 μmol/L), cells were collected and washed twice with phosphate buffered saline (PBS). The inhibitor group and the agonist group were treated as follows: After the HeLa cells was treated with 10 μmol/L GaAD19 for 5 h, 60 μmol/L SP600125 or 0.1 μmol/L Anisomycin were added to the treatment for 1 h. They were then fixed at 4 °C for 12–18 h in 70% ethanol which were precooled to −20 °C. Afterwards, cells were centrifuged again at 1 200 × *g* for 5 min, washed with pre-cooled PBS, stained with the cell cycle and apoptosis detection kit (Beyotime, Shanghai, China), and incubated at 37 °C for 30 min. The cell cycle was analyzed by flow cytometry (FACSCalibur, BD, USA).

#### 2.2.4 Apoptosis detection

HeLa cells were cultured in 6-well plates at a density of 4 × 10^5^ cells/well for 24 h in a 37 °C cell culture chamber with 5% CO_2_. After 6 h of treatment with different concentrations of GaAD19 (0, 5, 10 μmol/L), the cells were collected into flow cytometry tubes and washed twice with 4 °C precooled PBS. The inhibitor and agonist treatments were consistent with those in cell cycle analysis. The cells were then stained with the annexin V-fluorescein isothiocyanate/propidium iodide (annexin V-FITC/PI) apoptosis assay kit (Sangon Biotech, Shanghai, China). The stained cells were analyzed by flow cytometry (FACSCalibur, BD, USA).

#### 2.2.5 Terminal deoxynucleotidyl transferase-mediated dUTP nick end labeling (TUNEL) staining

A 24-well round coverslip was placed on a 6-well plate, and the cells at the logarithmic stage were inoculated on the 6-well plate at a density of 2 × 10^5^ cells/well. HeLa cells were treated with different concentrations of GaAD19 (0, 5, 10, 20 μmol/L) for 24 h and then fixed 12–18 h with 4% paraformaldehyde at 4 °C. Subsequently, apoptosis was detected using the Click-iT^TM^ TUNEL colorimetric kit (Thermo Fisher Scientific, Invitrogen, USA). All TUNEL stained cells were observed and photographed by confocal microscopy (ZEISS, Oberkochen, Germany).

#### 2.2.6 Immunocytochemical staining

A 24-well round coverslip was placed on a 12-well plate, and the cells at the logarithmic stage were inoculated on the 12-well plate at a density of 2 × 10^5^ cells/well. HeLa cells were treated with different concentrations of GaAD19 (0, 5, 10, 20 μmol/L) for 24 h and then fixed 5 h with 4% paraformaldehyde at 4 °C. To each well, 200 μL of iced acetone was added, and then kept at −20 °C for 7 min to permeabilize the cell membrane. Subsequently, the expression of Caspase-3 was detected using the Streptavidin Rabbit & Mouse Horseradish Peroxidase kit (CWBIO, Beijing, China) according to the instructions. All immunohistochemically stained cells were observed and photographed by confocal microscopy (ZEISS, Oberkochen, Germany).

### 2.3 Target prediction analysis

One hundred targets of GaAD19 were predicted using https://www.swisstargetprediction.ch/, and then https://cn.string-db.org/ was used for interaction network analysis. The obtained network was imported into Cytoscape (version 3.9.1) and six clusters were obtained through molecular complex detection (MCODE) module (conditions: Node Score Cutoff = 0.2, K-Core = 2). CytoHubba module analysis was performed on the highest ranked cluster to obtain Top10 genes.

### 2.4 Molecular docking

The structure of JNK (PDB ID, 4QTD) was obtained from the Protein Data Bank, and the structure of GaAD19 was drawn using ChemDraw 20.0 (CambridgeSoft, Cambridge, USA). Structures were prepared using AutoDockTools 1.5.6 (Molecular Graphics Laboratory, CA, Canada). The docking results with the lowest binding energy were plotted and analyzed using PyMOL software (The PyMOL Molecular Graphics System, Version 2.0 Schrödinger, LLC, New York, USA).

### 2.5 RNA extraction and quantitative real time polymerase chain reaction (qPCR) analysis

HeLa cells were treated with different concentrations of GaAD19 (0, 5, 10 μmol/L) for 2 h. Total RNA content was extracted using Trizol Reagent (Life, Waltham, USA). Total RNA was back-transcribed using a reverse transcription kit (Takara Bio, Shiga, Japan) to synthesize cDNA. The TB Green premix kit (Takara Bio) was used for real-ime PCR detection of cycle-related gene expression. The relative quantitative values of target genes were indicated by 2^–ΔΔCt^. All of the trials were carried out three times. Table 1 lists the primer sequences utilized in this research.

**Table 1:** Primer sequences.

Gene	Forward primer (5′−3′)	Reverse primer (3′−5′)
*CDK1*	AGAAGGTACTTACGGTGTGGT	GAGAGATTTCCCGAATTGCAGT
*CDK2*	CCTGCTTATCAATGCAGAGGG	TGCGGGTCACCATTTCAGC
*CDK4*	ATGGCTGCCACTCGATATGAA	TCCTCCATTAGGAACTCTCACAC
*CDK6*	GGCGTACCCACAGAAACCATA	AGGTAAGGGCCATCTGAAAACT
*CDK7*	ACTGTCCGGTGGAGGCATTA	CTGCTCTTTTCCGCTTTGTTG
*CDK8*	CGGGTCGAGGACCTGTTTG	TGCCGACATAGAAATTCCAGTTC
*Ccne1*	GTGGCTCCGACCTTTCAGTC	CACAGTCTTGTCAATCTTGGCA
*PCLAF*	ACCAAAGCAAACTACGTTCCA	TTTTCCCGACGAACTTGAAGAA
*Ube2c*	CTCCGCCTTCCCTGAGTCA	GGTGCGTTGTAAGGGTAGCC
*Crk*	GGAGACATCTTGAGAATCCGGG	ACGTAAGGGACTGGAATCATCC
*CrkL*	CGCTCCGCCTGGTATATGG	GGACACCGACAGCACATAGTC
*Shc*	TACTTGGTTCGGTACATGGGT	CTGAGTCCGGGTGTTGAAGTC
*GRB2*	CTGGGTGGTGAAGTTCAATTCT	GTTCTATGTCCCGCAGGAATATC
*Sos*	GAGTGAATCTGCATGTCGGTT	CTCTCATGTTTGGCTCCTACAC
*Ras*	ACAGAGAGTGGAGGATGCTTT	TTTCACACAGCCAGGAGTCTT
*Rac*	ATGTCCGTGCAAAGTGGTATC	CTCGGATCGCTTCGTCAAACA
*cdc42*	CCATCGGAATATGTACCGACTG	CTCAGCGGTCGTAATCTGTCA
*Gα12*	GGAGGGATTCTGGCATCAGG	CCGATCCGGTCCAAGTTGTC
*Gβ*	GTGAGCTTGACCAGTTACGG	TGTGATCTGAGAGAGAGTTGCAT
*18S*	GTAACCCGTTGAACCCCATT	CCATCCAATCGGTAGTAGCG

### 2.6 Western blot analysis

HeLa cells were treated with different concentrations of GaAD19 for 24 h. Cells or tumor tissues were lysed in RIPA lysate (CWBIO, Beijing, China): protease inhibitor: phosphatase inhibitor = 100 μL:1 μL:1 μL, respectively. Cell lysates were centrifuged after ultrasound (12 000 r/min at 4 °C, 30 min) and then mixed with 5 × protein loading buffer (CWBIO, Beijing, China) and boiled at 100 °C for 10 min to facilitate protein denaturation.

Protein samples were isolated using 10%−12% sodium dodecyl sulfate–polyacrylamide gel electrophoresis (SDS-PAGE) and then transferred to 0.45 μm nitrocellulose (NC) membrane (Cytiva, New York, USA), which was sealed in 5% skim milk for 2 h to block non-specific antibody binding. This was then incubated for 12–18 h with suitably diluted primary antibody at 4 °C. The membrane was washed with tris buffered saline + Tween-20 (TBST) thrice, followed by incubation with secondary antibody diluted at 1:2 000 at room temperature (about 25 °C) for 1 h. Enhanced chemiluminescence-A (ECL-A): enhanced chemiluminescence-B (ECL-B) color solution (CWBIO, Beijing, China) = 1:1 was mixed and incubated for 5 min at room temperature (about 25 °C) away from light. Images were taken using the S Bio-RAD imaging system (Bio-RAD, Hercules, CA, USA), and the development results were analyzed using ImageJ software. β-actin was used as an internal reference. All the primary antibodies and dilutions used in this study are listed in Table 2.

**Table 2:** Primary antibodies.

Antibody	Company	Cat	Dilution
BCl_2_	Abcam	ab182858	1:500
Bax	Proteintech	50599–2-Ig	1:1 000
Caspase-9	ABclonal	A0281	1:500
p-AKT	Proteintech	66444–1-Ig	1:200
AKT	Proteintech	10176–2-AP	1:1 000
p-p38 MAPK	Cell signaling	4511	1:500
p38 MAPK	Proteintech	14064–1-AP	1:500
p-JNK	Proteintech	80024–1-RR	1:1 000
JNK	Proteintech	24164–1-AP	1:2 000
p-AMPK*α*1-S485	ABclonal	AP0871	1:2 000
p-AMPK*α*1-S496	ABclonal	AP1002	1:2 000
AMPK *α*1	Proteintech	10929–2-AP	1:200
p-AMPK*β*1-S108	ABclonal	AP0597	1:200
AMPK *β*1	Proteintech	10308–1-AP	1:500
p-ERK	ABclonal	RK05775	1:500
ERK	Proteintech	16443–1-AP	1:1 000
MAP2K4	Proteintech	17340–1-AP	1:1 000
MAP2K7	Proteintech	55030–1-AP	1:500
Cleaved PARP1	ABclonal	A19612	1:2 000
Caspase-3	Proteintech	66470–2-Ig	1:300
*β*-Actin	Abcam	ab8226	1:2 000

**Note:** Bcl-2, B-cell lymphoma-2; Bax, Bcl-2-associated X; p-AKT, phosphorylated-protein kinase B; AKT, protein kinase B; p-p38 MAPK, phosphorylated-p38 mitogen activated protein kinases; p38 MAPK, p38 mitogen activated protein kinases; p-JNK, phosphorylated-c-Jun *N*-terminal kinase; JNK, c-Jun *N*-terminal kinase; p-AMPK*α*1-S485, phosphorylated-adenosine 5′-monophosphate (AMP)-activated protein kinase *α*1-S485; p-AMPK*α*1-S496, phosphorylated-adenosine 5′-monophosphate (AMP)-activated protein kinase *α*1-S496; AMPK *α*1, adenosine 5′-monophosphate (AMP)-activated protein kinase *α*1; p-AMPK*β*1-S108, phosphorylated-adenosine 5′-monophosphate (AMP)-activated protein kinase *β*1-S108; AMPK *β*1, adenosine 5′-monophosphate (AMP)-activated protein kinase *β*1; p-ERK, phosphorylated-extracellular signal-regulated kinase; ERK, extracellular signal-regulated kinase; MAP2K4, mitogen-activated protein kinase kinase 4; MAP2K7, mitogen-activated protein kinase kinase 7; cleaved PARP1, cleaved poly(ADP-ribose) polymerase 1; *β*-Actin, beta-actin.

### 2.7 Animal model, treatments, and histology

Six-week female BALB/c nude mice (18 ± 2) g were purchased from Vital River Laboratory (Beijing, China, certificate no. 110011221112424912). This work was approved by the Institutional Animal Care and Use Committee (Chinese Academy of Medical Sciences and Peking Union Medical College, Beijing, China, SLXD −20220317028). The blank group was not inoculated with tumor, and 30 mice in the other six groups were subcutaneously injected with a 0.1 mL U14 cell suspension of 8 × 10^6^ cells/mL in the right axilla. When the tumor volume reached about 60 mm^3^, 30 mice were randomly divided into six groups with five mice in each group. Corresponding treatments were given: 1) Model group: Mice were intraperitoneal injected with saline every day. 2) Low dose GaAD19 group: Mice were intraperitoneally injected with GaAD19 (25 mg/kg) every day. 3) Medium dose GaAD19 group: Mice were intraperitoneally injected with GaAD19 (50 mg/kg) every day. 4) High dose GaAD19 group: Mice were intraperitoneally injected with GaAD19 (100 mg/kg) every day. 5) Cyclophosphamide (CTX) group: Mice were intraperitoneally injected with CTX (25 mg/kg) every day. 6) Medium dose GaAD19 + Anisomycin group: Mice were intraperitoneally injected with GaAD19 (50 mg/kg) + Anisomycin (5 mg/kg) every day. The blank group was intraperitoneally injected with saline daily. Weight and tumor volume were measured every two days. The tumor volume was calculated as follows: Tumor volumes (V) = 1/2 × length × width × width.

The mice were sacrificed 14 d after modeling, and blood was collected for ELISA detection. Liver, kidney, spleen, and tumor tissues were collected and stored at −80 °C in 4% tissue fixation solution (Coolaber, Beijing, China). The tumor tissues were processed for western blot experiment. The liver, kidney, spleen and tumor tissues were processed for hematoxylin and eosin (H&E).

### 2.8 Cytokine enzyme-linked immune sorbent assay (ELISA)

The animal orbital blood was collected in a 1.5 mL centrifuge tube, left at room temperature for 4 h, centrifuged at 3 000 r/min for 15 min, and then the upper serum was carefully absorbed and transferred to a new 1.5 mL centrifuge tube. Serum interleukin-2 (IL-2) and IL-10 levels were measured according to the ELISA kit instructions (Solarbio, Beijing, China).

### 2.9 Statistical analysis

SPSS 25 for Windows was used for data analyses. All results were expressed as means ± SD. The differences between groups were analyzed by One-way analysis of variance (ANOVA) and post-hoc comparisons between any two groups were made using the least significant difference (LSD) test. At *P* < 0.05, the data was considered statistically significant.

## 3 Results

### 3.1 Synthesis of GaAD19 by structural modification from GaA

**Fig. 1:**
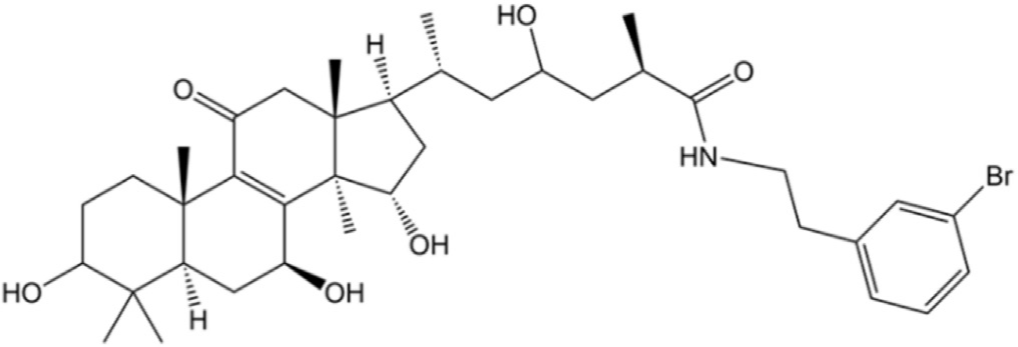
Chemical structure of GaAD19.

GaAD19 (Fig. 1) was synthesized according to the amidation and reduction procedures. Amidation of C-26 carboxylic acid of GaA: ^1^H NMR (600 MHz, CDCl_3_) *δ*: 7.36–7.35 (m, 2H), 7.18 (t, *J* = 8.0 Hz, 1H), 7.13 (d, *J* = 7.7 Hz, 1H), 5.97 (t, *J* = 5.0 Hz, 1H), 4.78 (t, *J* = 7.9 Hz, 1H), 4.61 (dd, *J* = 9.6, 6.8 Hz, 1H), 3.45 (dt, *J* = 13.3, 7.0 Hz, 2H), 2.88–2.81 (m, 3H), 2.78–2.73 (m, 1H), 2.67 (ddd, *J* = 7.0, 4.4, 2.0 Hz, 1H), 2.50–2.47 (m, 3H), 2.42–2.38 (m, 2H), 2.22 (dd, *J* = 16.2, 9.5 Hz, 1H), 2.04–2.02 (m, 2H), 1.81–1.80 (m, 3H), 1.71–1.68 (m, 2H), 1.49–1.44 (m, 1H), 1.27 (s, 3H), 1.25 (s, 3H), 1.11 (s, 3H), 1.09 (s, 3H), 0.99 (s, 3H), 0.87 (d, *J* = 6.4 Hz, 3H). ^13^C NMR (151 MHz, CDCl_3_) *δ*: 217.27, 209.50, 199.63, 175.81, 159.47, 141.23, 140.11, 131.88, 130.17, 129.65, 127.46, 122.58, 72.27, 68.79, 60.44, 53.99, 51.73, 49.83, 48.75, 48.12, 47.08, 46.76, 46.62, 40.50, 37.97, 36.22, 35.96, 35.52, 35.26, 34.29, 32.74, 28.93, 27.36, 20.70, 19.66, 19.53, 19.39, 17.95, 17.29, 14.18. ESI-HRMS: *m*/*z* calcd for (M + K) C_38_H_52_O_6_NBrK, 738.259 5; found, 738.257 8. Reduction of C-3 ketone: ^1^H NMR (500 MHz, MeOD) *δ*: 7.44 (s, 1H), 7.39 (s, 1H), 7.24 (s, 2H), 4.79–4.76 (m, H), 4.57–4.54 (m, 1H), 3.72–3.70 (m, 1H), 3.56–3.45 (m, 1H), 3.35(m, 1H), 3.20–3.17 (m, 1H), 2.90–2.73(m, 4H), 2.57–2.43 (m, 2H), 2.14 (dd, *J* = 11.6, 7.4 Hz, 1H), 1.87–1.86(m, 3H), 1.71–1.63(m, 4H), 1.56–1.52(m, 3H), 1.29 (s, 3H), 1.27 (s, 3H), 1.12–1.10 (m, 3H), 1.05 (s, 3H), 1.10–0.96 (m, 6H), 0.91–0.87 (m, 3H). ^13^C NMR (151 MHz, MeOD) *δ*: 201.16 (s), 201.07 (s), 178.51 (s), 177.97 (s), 159.97 (s), 77.56 (s), 71.93 (s), 68.82 (s), 66.96 (s), 66.22 (s), 44.37 (s), 44.30 (s), 42.40 (s), 40.53 (s), 40.10 (s), 40.01 (s), 38.31 (s), 37.59 (s), 37.02 (s), 35.95 (s), 35.74 (s), 34.76 (s), 34.64 (s), 34.54 (s), 33.98 (s), 32.45 (s), 27.61 (s), 27.35 (s), 27.02 (s), 18.61 (s), 18.50 (s), 17.84 (s), 17.73 (s), 16.28 (s), 16.20 (s), 15.03 (s). ESI-HRMS: *m*/*z* calcd for (M + H) C_38_H_57_BrNO_6_, 702.336 9; found,702.335 6.

The NMR and HRMS results (Fig. S1–S3) proved that two ketones (C-3 and C-26) of the structure were both converted into hydroxyl. The product obtained was used as a mixture of optical isomers without further purification.

### 3.2 GaAD19 inhibited proliferation of HeLa and SiHa cells

**Fig. 2:**
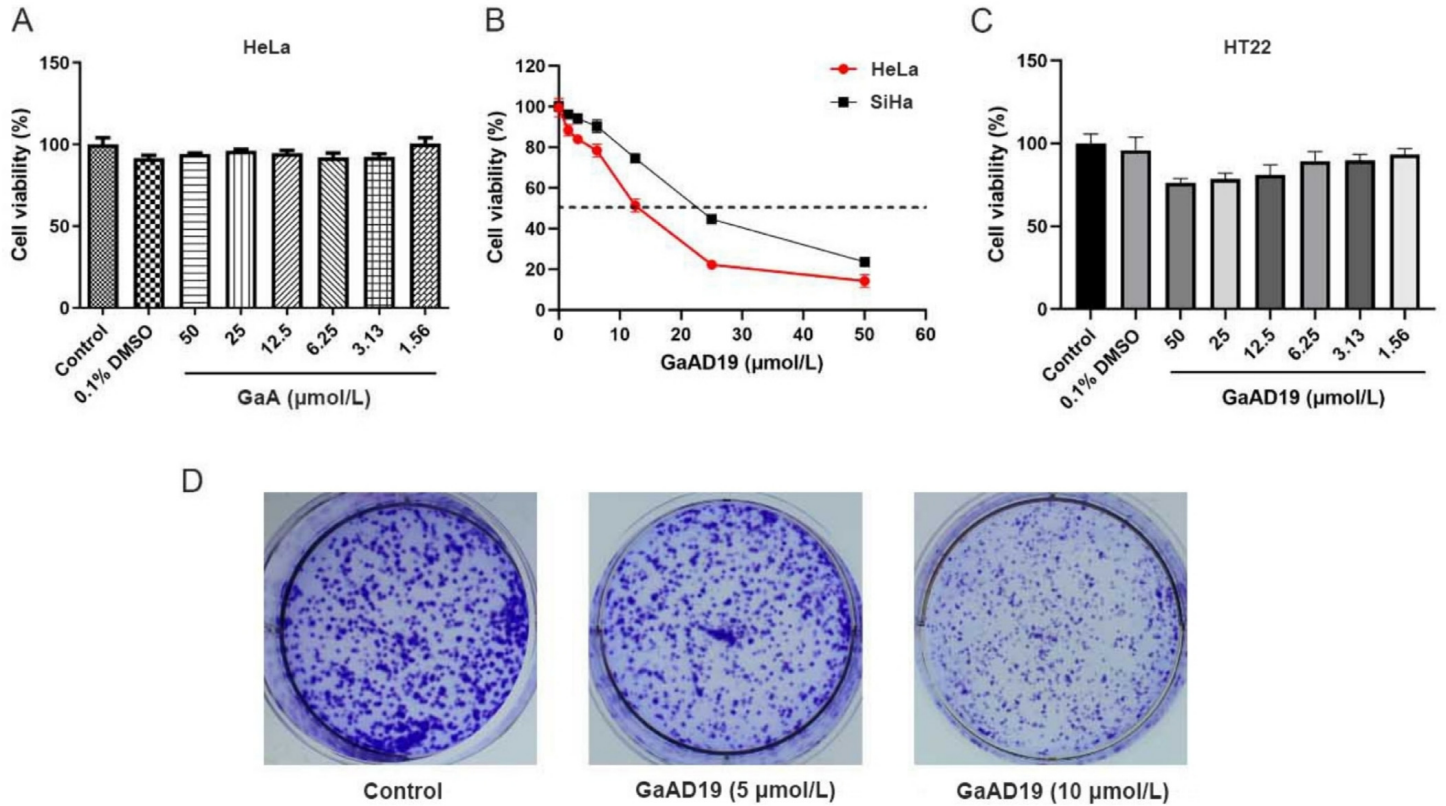
Growth inhibition of cells under GaAD19 or GaA treatment (means ± SD, *n* = 3). (A) Cell viability of HeLa cells treated with GaA for 48 h. (B) Dose-response profile of effects of GaAD19 on viability of HeLa and SiHa cells. (C) Cell viability of HT22 cells treated with GaAD19 for 48 h. (D) GaAD19 inhibits colony formation of HeLa cells.

The cytotoxic effect of GaA on Hela cells and GaAD19 on human cervical cancer cells, human liver cancer cells, human breast cancer cells and other nine tumor cell lines were studied using a CCK-8 assay (Table S1). The results showed that GaA had weak antitumor activity (Fig. 2A). The viability of the HeLa cells showed a strong dose dependent inhibition by GaAD19, its IC_50_ value is 11.20 μmol/L, such that the inhibition increased as the dosing concentration increased (Fig. 2B). For example, 6.25 μmol/L GaAD19 inhibited 16% of the HeLa cells viability, whereas 12 μmol/L GaAD19 altered approximately 50% of the HeLa cell viability. GaAD19 showed weak cytotoxicity to hippocampal neuronal (HT22) cell line (Fig. 2C). The results of colony formation experiments showed that 10 μmol/L GaAD19 significantly inhibited the proliferation of HeLa cells (Fig. 2D).

### 3.3 GaAD19 induced apoptosis of HeLa cells

**Fig. 3:**
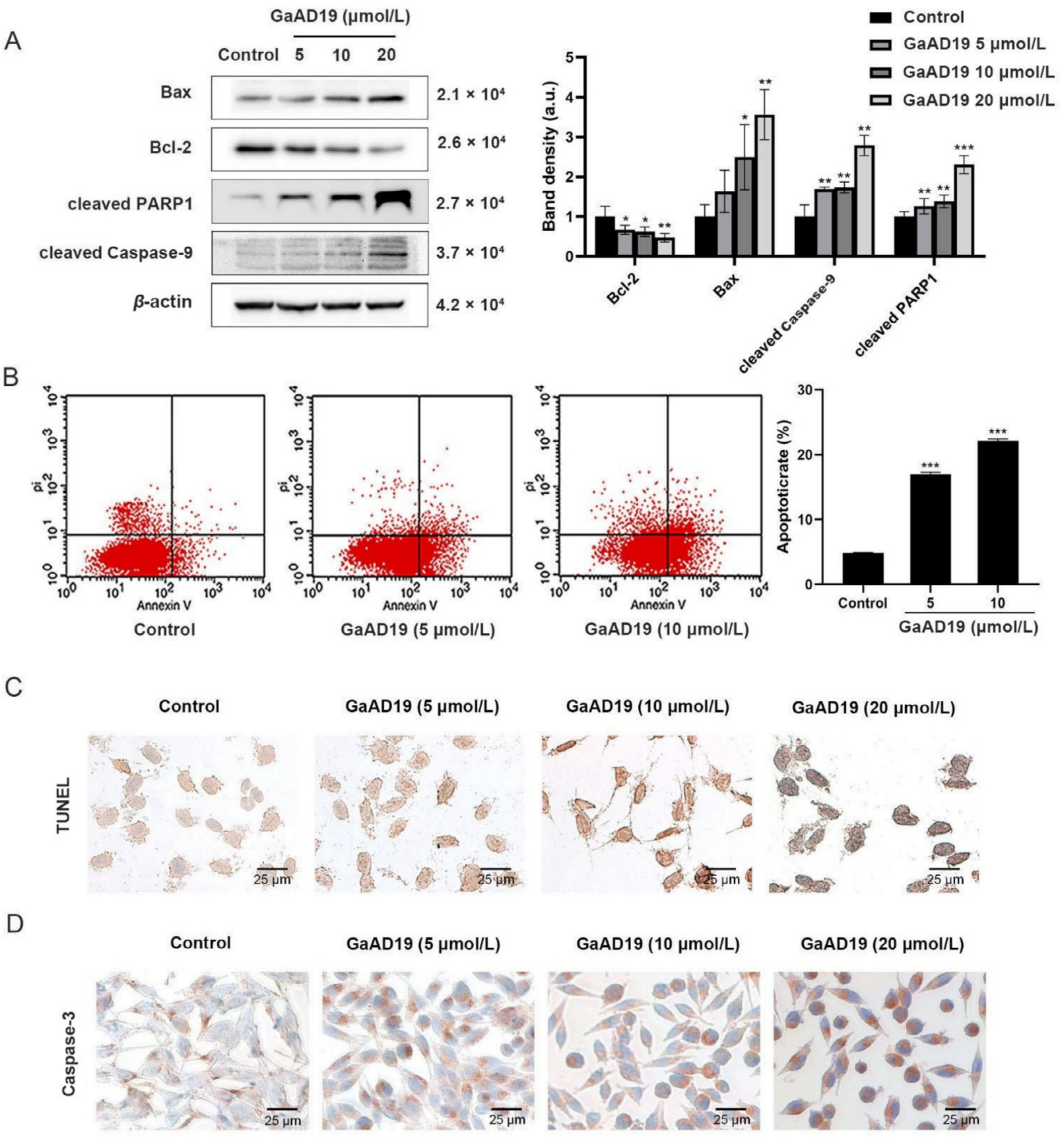
GaAD19 induces HeLa cells apoptosis (means ± SD, *n* = 3). (A) Western blot analysis of HeLa cells with GaAD19 treatment for 24 h on expression of Bax, Bcl-2, PARP1, Caspase-9, and other proteins. (B) Annexin V-FITC/PI staining to detect apoptosis-inducing effect of GaAD19 on HeLa cells. (C) HeLa cell apoptosis was detected by TUNEL staining (× 400, scale bars: 25 μm). (D) Expression of Caspase-3 in HeLa cells was detected by immunohistochemical staining (× 400, scale bars: 25 μm). (*P < 0.05, ^**^P < 0.01, ^***^P < 0.001 *vs* control group).

Apoptosis is a key natural process that involves programmed cell death, and thus removes unwanted cells. Abnormal production of apoptotic proteins disrupts the balance between cell proliferation and apoptosis, and is reported lead to cancer development (Fesik, 2005). To evaluate the inhibitory effect of GaAD19 on the growth of HeLa cells, we assessed the expression of apoptosis regulatory proteins Bcl-2, Bax, cleaved PARP1, and cleaved Caspase-9 using western blot. As shown in Fig. 3A, Bax, cleaved PARP1, and cleaved Caspase-9 protein levels were significantly increased in HeLa cells that were treated with GaAD19 for 24 h. In addition, immunohistochemical staining showed that GaAD19 increased the number of Caspase-3-positive cells in HeLa cells (Fig. 3D). Further, flow cytometry analyses showed that a 6-h exposure of GaAD19 had strong apoptotic-inducing activity on HeLa cells. As shown in Fig. 3B, the percentage of apoptotic cells increased from 4.86% to either 16.63% or 22.45% after cells were treated with 5 or 10 μmol/L GaAD19, respectively. These data suggested that GaAD19 activated Bax and cleaved Caspase-9 in HeLa cells in a dose-dependent manner to induce apoptosis. TUNEL staining results (Fig. 3C) further supported this interpretation. These data suggested that GaAD19 induced apoptosis of HeLa cells in a dose-dependent manner.

### 3.4 GaAD19 regulated HeLa cell cycle

**Fig. 4:**
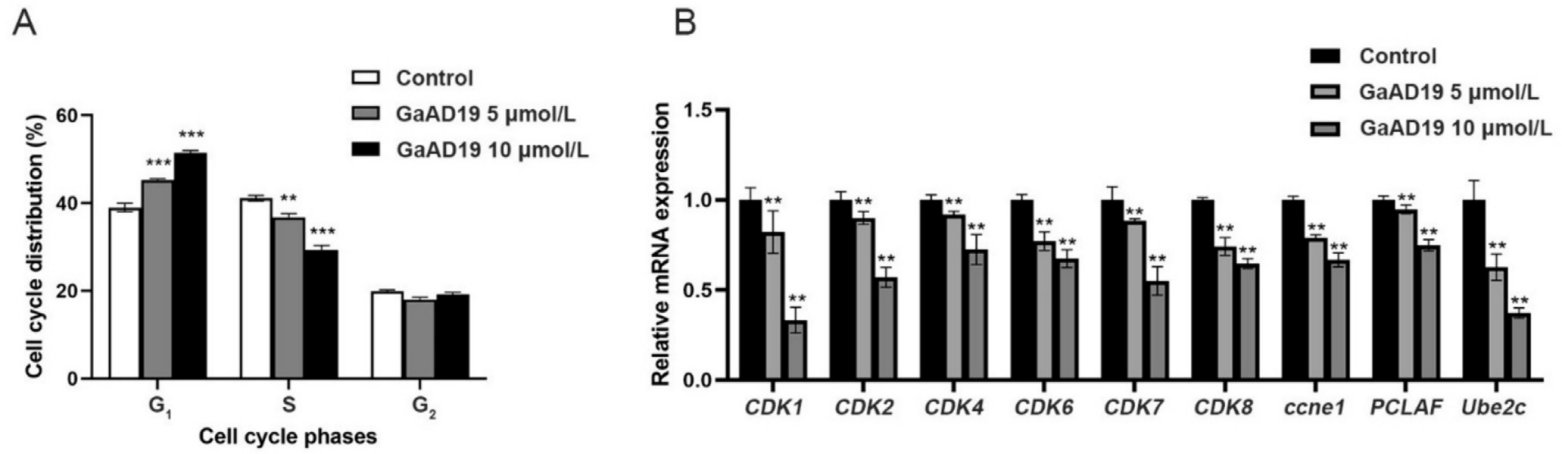
Effects of GaAD19 on HeLa cell cycle (means ± SD, *n* = 3). (A) Effect of GaAD19 on cell cycle distribution of HeLa cells. (B) Effect of GaAD19 on mRNA level of cell cycle regulatory protein in HeLa cells was determined by qPCR. (^**^P < 0.01 *vs* control group).

The cell cycle is an ordered set of events that occur in a cell, causing it to grow and divide into daughter cells (Schafer, 1998). The typical cell cycle comprises four main phases: G_1_, S, G_2_, and M. The main purpose of studying the cell cycle is to infer the distribution of cells at various stages (Wang, 2021). To assess this, we used flow cytometry analyses and evaluated the effect of GaAD19 on the HeLa cell cycle. In a dose-dependent manner, GaAD19 greatly enhanced the G_1_ phase cell population while decreasing the S phase cell population (Fig. 4A). Cell population in the G_1_ phase increased from 37.96% (control) to 45.09% and 51.76% under 5 μmol/L and 10 μmol/L GaAD19 treatment, respectively. Further, S phase cell population decreased from 41.77% (control) to 35.8% and 30.47%, respectively.

Next, to evaluate the effect of GaAD19 on HeLa cell cycle-related gene expression levels, we detected the expression of a few related genes using qPCR. As indicated in Fig. 4B, GaAD19 significantly decreased the expression of *CDK1*, *CDK2*, *CDK4*, *CDK6*, *CDK7*, *CDK8*, *ccne1*, *PCLAF*, and *Ube2c*.

### 3.5 GaAD19 targeted and suppressed the JNK pathway

**Fig. 5:**
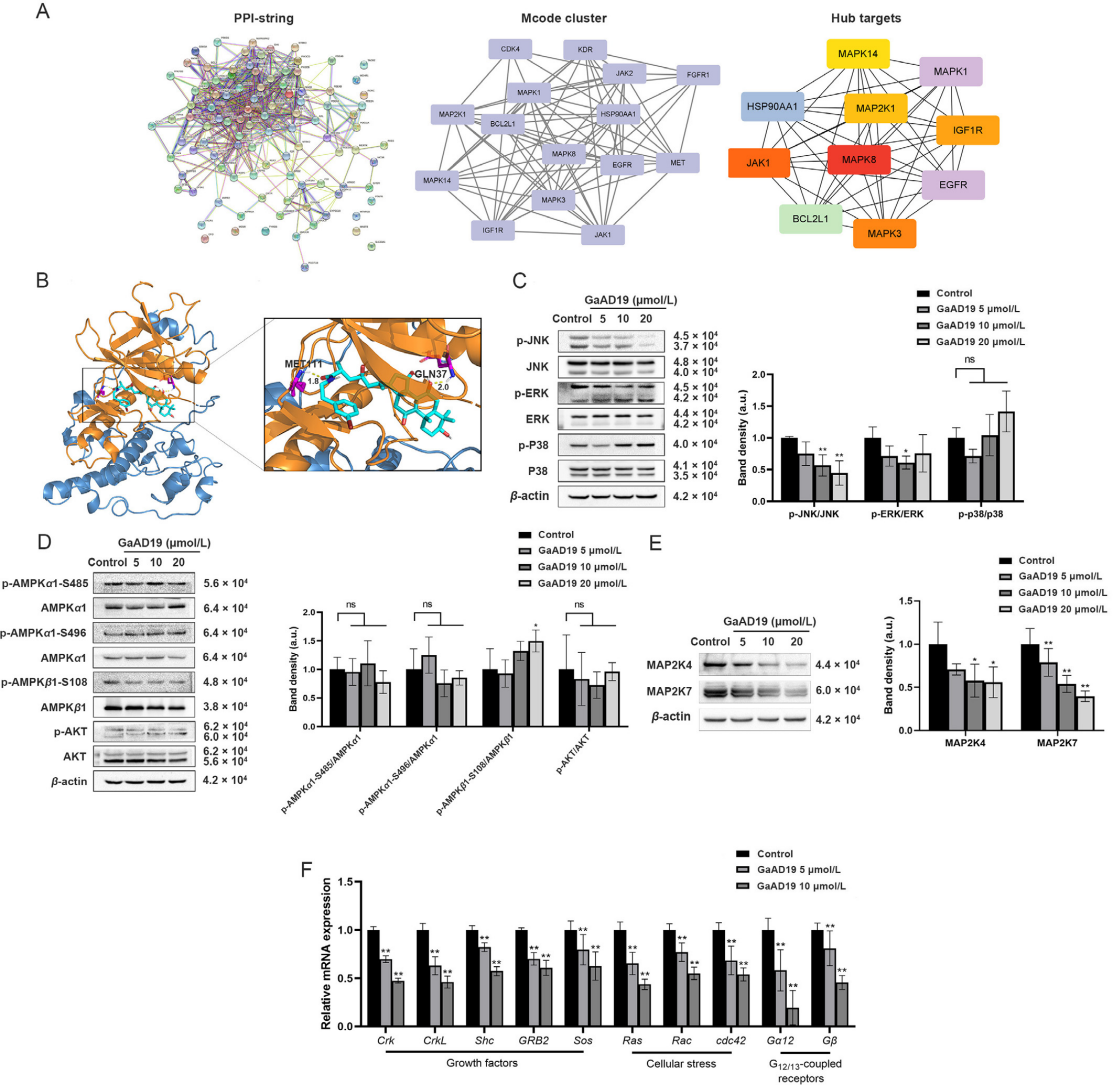
(A) Target prediction analysis results. (B) Prediction of GaAD19-JNK interaction. (C–E) Western blot analysis of HeLa cells with GaAD19 treatment for 24 h on expression of JNK, p-JNK, ERK, p-ERK, p38, p-p38 (C), AMPK *α*1, p-AMPK *α*1-S485, p-AMPK *α*1-S496, AMPK *β*1, p-AMPK *β*1-S108, Akt, p-Akt (D), MAP2K4, MAP2K7 (E), and other proteins (means ± SD, *n* = 3). (F) Effect of GaAD19 on mRNA level of JNK pathway membrane protein receptor in HeLa cells was determined by qPCR (means ± SD, *n* = 3). (^**^P < 0.01, ^***^P < 0.001 *vs* control group, ns indicates no significant difference).

The MAPK pathway plays an important role in various cell functions and regulates many important physiological effects such as cell growth, differentiation, stress, and metastasis. The MAPK signaling pathway is closely related to cancer formation (Wagner and Cancer, 2009, Zhou et al., 2020). Through target prediction analysis, it was found that *MAPK8* is a hub gene (Fig. 5A). A total of 100 dockings were performed on JNK and GAaD19, and the lowest free energy of binding (FEB) obtained was −51.51 kJ/mol. The possible hydrogen bond length between GaAD19 and MET111 was 1.8 Å, while it was 2.0 Å between GaAD19 and GLN37 (Fig. 5B). Western blot was used to investigate the effects of GaAD19 on various proteins involved in the MAPK, AMPK and PI3K/Akt pathways in HeLa cells. As demonstrated in Fig. 5C and D, the expression of phosphorylated JNK decreased with an increased concentration of GaAD19, while no significant difference in the expression of p-ERK, p-p38, p-AMPK *α*1-S485, p-AMPK *α*1-S496, p-AMPK *β*1-S108 and p-Akt was observed. As shown in Fig. 5E, the expression of MAP2K4 and MAP2K7, the upstream activators of JNK, were inhibited with an increased concentration of GaAD19. These data suggested that GaAD19 may inhibit the JNK signaling pathway in HeLa cells. In order to further identify the receptors that GaAD19 is binding, inducing inhibition of JNK pathway, we used qPCR to detect the expression of four kinds of membrane protein receptors: growth factors, cellular stress, Fas ligand (FasL), and G_12/13_-coupled receptors. As shown in Fig. 5F, GaAD19 mainly acted on growth factors, cellular stress, and G_12/13_-coupled receptors membrane protein receptors, reducing the expression of *Crk*, *CrkL*, *Shc*, *GRB2*, *Sos*, *Ras*, *Rac*, *cdc42*, *Gα 12*, and *Gβ*, and thus inhibiting the JNK pathway.

### 3.6 Effects of GaAD19 on cell proliferation and cell cycle of HeLa cells were abolished and reinforced by Anisomycin and SP600125, respectively

**Fig. 6:**
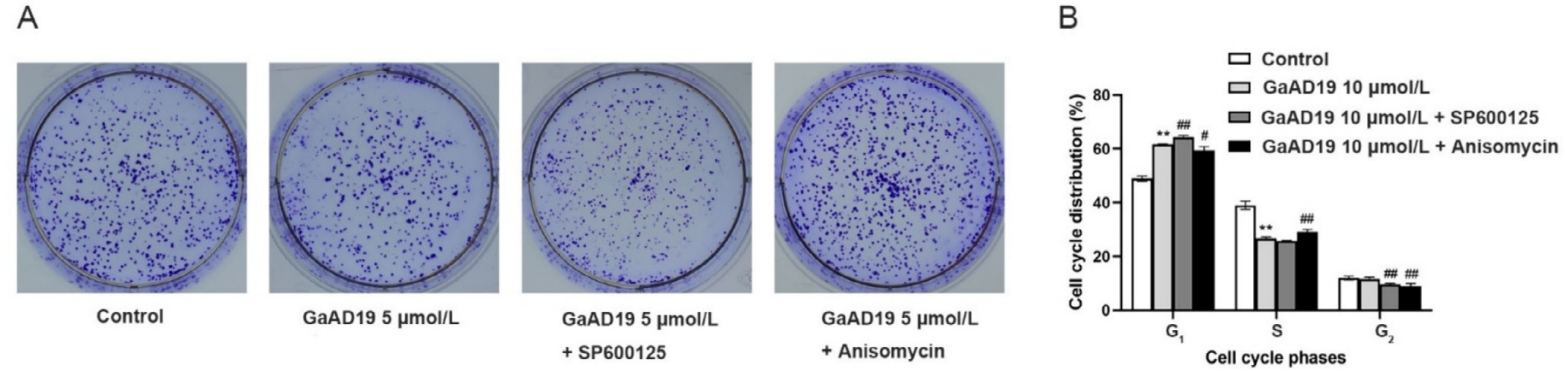
Effects of GaAD19 combined with SP600125 and Anisomycin on cell proliferation and cell cycle of HeLa cells (means ± SD, *n* = 3). (A) Anisomycin reversed anti-proliferative activity of GaAD19, and SP600125 promoted anti-proliferative activity of GaAD19. (B) Anisomycin reversed cycle blocking activity of GaAD19, and SP600125 promoted cycle blocking activity of GaAD19. (*P < 0.05, ^**^P < 0.01 *vs* control group; ^#^*P* < 0.05, ^##^*P* < 0.01 *vs* GaAD19 group).

To verify the hypothesis that GaAD19 may inhibit cell proliferation and block the cell cycle through the JNK pathway, we treated HeLa cells with GaAD19 in combination with a JNK inhibitor SP600125 and JNK agonist Anisomycin. As indicated in Fig. 6A, the number of cell colonies in the SP600125 + GaAD19 group was lower than the number of colonies in the 5 μmol/L GaAD19 group. Further, the colony number of Anisomycin + GaAD19 group was more than that of the 5 μmol/L GaAD19 group. Flow cytometry results (Fig. 6B) showed that the 10 μmol/L GaAD19 group blocked the G_1_ phase cell cycle and increased the G_1_/S ratio, while the SP600125 + GaAD19 group further blocked the G_1_ phase cell cycle and continued to increase the G_1_/S ratio. The Anisomycin + GaAD19 group antagonized this effect and reduced the G_1_/S ratio.

### 3.7 Effects of GaAD19 on HeLa cell apoptosis were abolished and reinforced by Anisomycin and SP600125, respectively

**Fig. 7:**
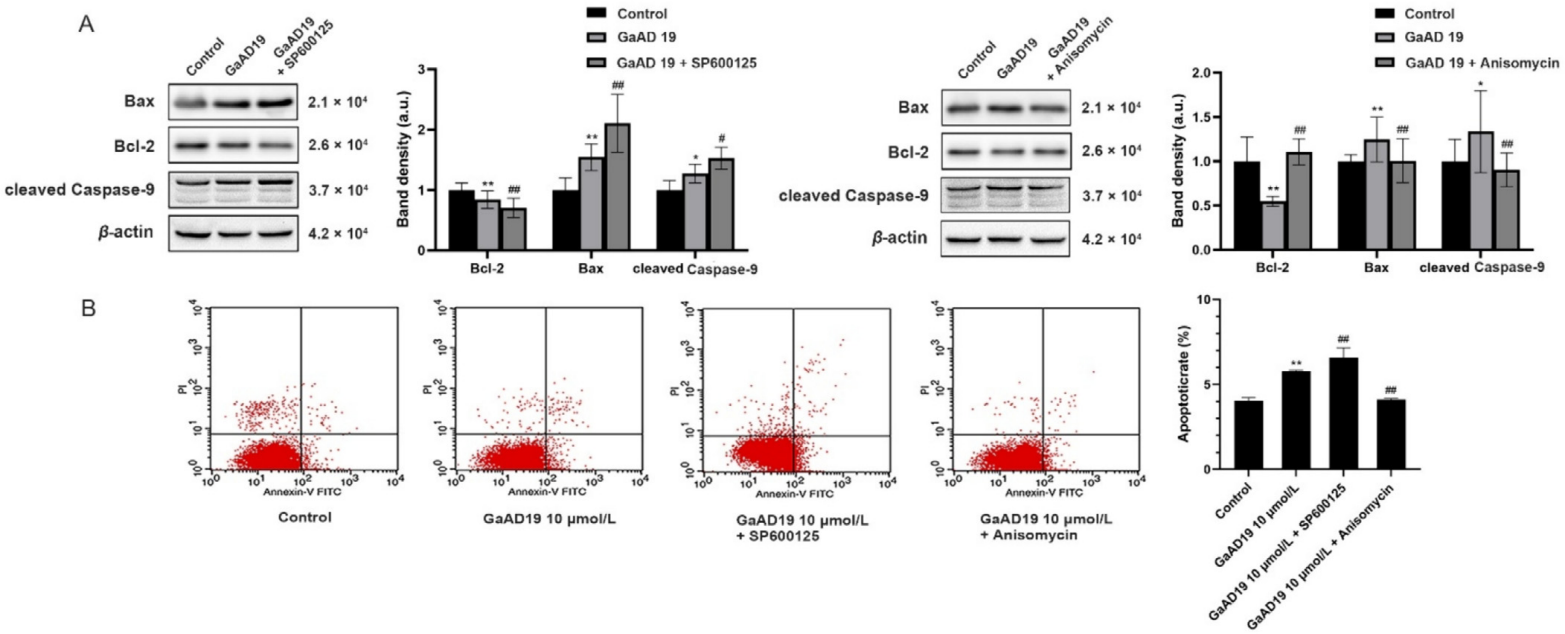
Effects of GaAD19 combined with SP600125 and Anisomycin on apoptosis of HeLa cells (means ± SD, *n* = 3). (A) Western blot analysis of HeLa cells with GaAD19 combined with SP600125 or Anisomycin treatment for 24 h on expression of Bax, Bcl-2, Caspase-9, and other proteins. (B) Annexin V-FITC/PI staining to detect apoptosis-inducing effect of GaAD19 in combination with either SP600125 or Anisomycin on HeLa cells. (*P < 0.05, ^**^P < 0.01 *vs* control group; ^#^*P* < 0.05, ^##^*P* < 0.01 *vs* GaAD19 group).

To investigate whether inhibition of the JNK pathway causes HeLa cell apoptosis, we conducted a series of experiments. As shown in Fig. 7A, Western blot results showed that the 10 μmol/L GaAD19 group induced down-regulation of Bcl-2 and up-regulation of Bax and cleaved Caspase-9. Importantly, application of the SP600125 + GaAD19 group further promoted this effect. Anisomycin treatment reversed the effects of GaAD19 on the expression of Bcl-2, Bax, and cleaved Caspase-9 proteins in HeLa cells. As shown in Fig. 7B, after cells were treated with 10 μmol/L GaAD19, the percentage of apoptotic cells increased from 3.92% to 5.69%. When GaAD19 was combined with SP600125, the percentage of apoptotic cells increased to 7.20%. However, after the combination of GaAD19 and Anisomycin, the percentage of apoptotic cells decreased to 4.09%. These data confirmed that GaAD19 induced HeLa cell apoptosis by inhibiting the JNK signaling pathway.

### 3.8 GaAD19 inhibited tumor growth in U14 cervical cancer mice

**Fig. 8:**
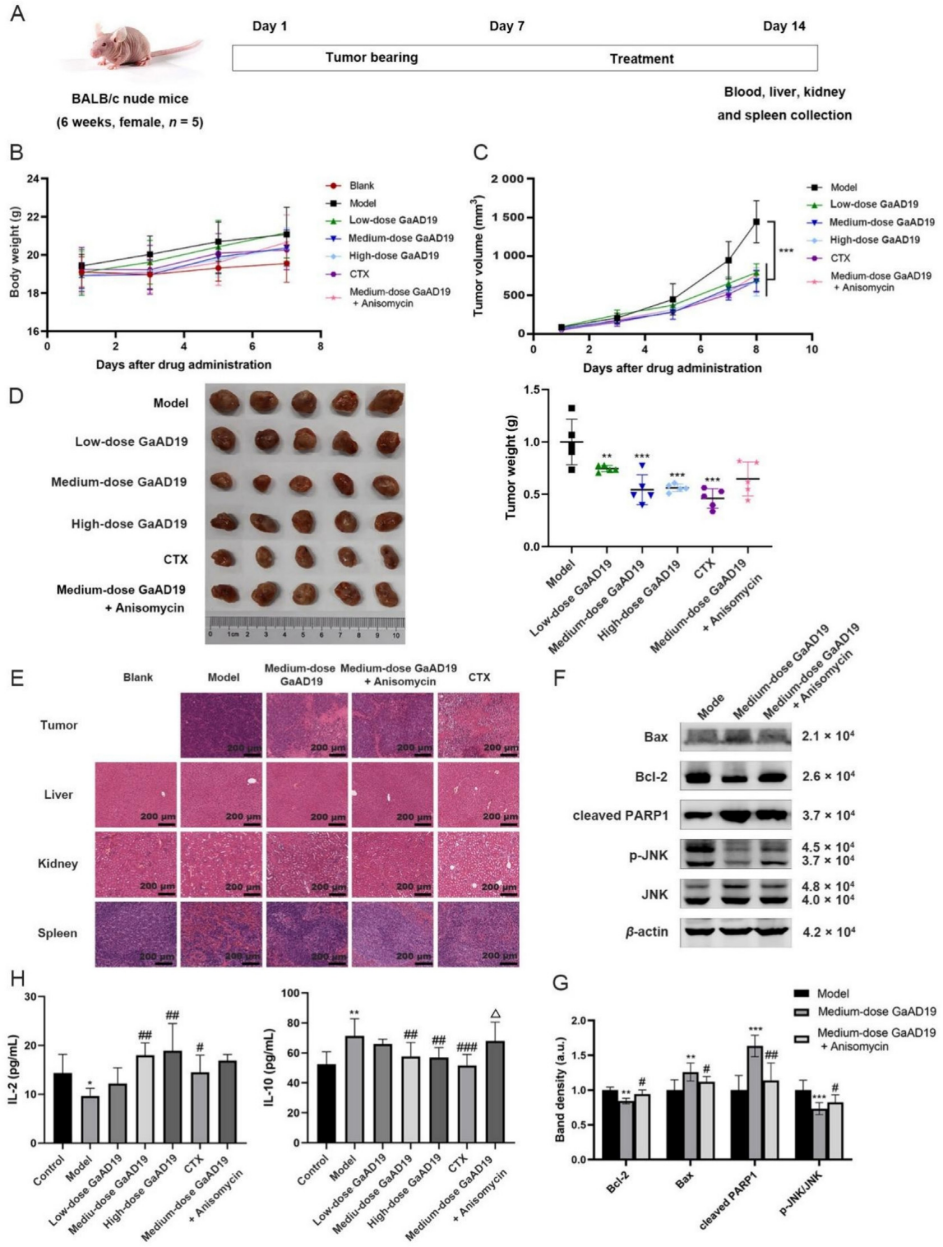
GaAD19 inhibited tumor growth in U14 cervical cancer mice. (A) Animal experiment program. (B–C) Changes in body weight (B) and tumor volume (C) of nude mice (means ± SD, *n* = 5). (D) Image of tumor tissue and tumor weight analysis (means ± SD, *n* = 5). (E) Hematoxylin & eosin staining of tumor, liver, kidney and spleen in nude mice (means ± SD, *n* = 3) (× 20, scale bars: 200 μm). (F) Effects of GaAD19 on expression of Bax, Bcl-2, PARP1, p-JNK, and JNK in tumor tissues of U14 xenograft nude mice were detected by Western blot (means ± SD, *n* = 3). (G). Statistical analysis of Western blot results (means ± SD, *n* = 3). (*P < 0.05, ^**^P < 0.01, ^***^P < 0.001 *vs* model group; ^#^*P* < 0.05, ^##^*P* < 0.01 *vs* medium-dose group). (H) Effect of GaAD19 on IL-2 and IL-10 in nude mice serum (means ± SD, *n* = 5). (*P < 0.05, ^**^P < 0.01 *vs* control group; ^#^*P* < 0.05, ^##^*P* < 0.01, ^###^*P* < 0.001 *vs* model group; ^△^*P* < 0.05 *vs* medium-dose group).

Through the establishment of HeLa and U14 cervical cancer xenograft models, we found that human cervical cancer HeLa cells were difficult to form solid tumors in nude mice, and it has been reported that the tumor formation of HeLa cells in nude mice seems to be controversial (Dirks, 2021). Therefore, we chose mouse cervical cancer U14 cells to construct a nude mouse model of cervical cancer. To evaluate the inhibitory effect of GaAD19 on tumor growth of U14 cervical cancer mice, we established a subcutaneous cervical cancer xenograft model and treated nude mice with different doses of GaAD19, CTX, or medium dose GaAD19 combined with a JNK agonist (Fig. 8A). At the beginning of the experiment, the mice in each group had normal behavior and mental state. At the later stage of the experiment, the behavior and mental states of the mice in each dose group of GaAD19 were better than those in the model group. In the CTX group, the activities of drinking water and eating decreased, and the body weight decreased significantly compared with the model group. The mice in the blank group were normal in all aspects (Fig. 8B). Compared with the model group, the tumor volume and tumor weight of mice in each dose group of GaAD19 were significantly reduced. At the same time, the tumor volume and tumor weight of the mice in the medium dose GaAD19 combined with JNK agonist group were slightly higher than those in the medium dose GaAD19 group (Fig. 8C and D). Western blot results showed that GaAD19 promoted tumor cell apoptosis by inhibiting the JNK pathway (Fig. 8F and G). H&E staining (Fig. 8E) provided additional evidence that GaAD19 promoted tumor cell necrosis after treatment, and had low toxic and side effects on normal tissues. ELISA results showed that compared with the control group, the serum IL-2 content in the model group was significantly decreased and IL-10 content was significantly increased. Compared with the model group, the serum IL-2 content in the GaAD19 low, medium and high dose groups and the CTX group was significantly increased, while the IL-10 content was significantly decreased. Compared with the GaAD19 medium-dose group, the serum IL-2 content in the combined treatment group was slightly decreased, and the IL-10 content was significantly increased (Fig. 8H). These results suggested that GaAD19 inhibited the tumor growth of U14 cervical cancer mice by inhibiting the JNK pathway.

## 4 Discussion

GaAD19 is a compound derived from the structural modification of GaA. Previous studies have reported that GaA inhibits the proliferation, migration and invasion of various immortal cell lines, namely HeLa, HepG2, MDA-MB-231 (Yang et al., 2018, Yao et al., 2012), and other tumor cells. However, the antitumor mechanism of GaAD19 on HeLa cells is poorly defined. The results showed that GaAD19 had strong cytotoxicity to HeLa cells, and its IC_50_ value (50% inhibitory concentration) was significantly lower than that of other cell lines. In colony formation experiments, different concentrations of GaAD19 can significantly inhibit the proliferation activity of HeLa cells. Further, GaAD19 led to cell cycle arrest in the G_1_ phase, affected the normal replication of S phase DNA, inhibited the synthesis of DNA, and thus hindered the growth of HeLa cells.

Cyclin-dependent kinases (CDK) family proteins are involved in orderly cell cycle events, and play an important role in cell cycle regulation (Zhang et al., 2021). Consistent with this role, this study demonstrated that GaAD19 application decreased the mRNA expression level of Ccne1 (a cyclin family protein) and regulated cell progression from G_1_ phase to S phase, and that its activity was necessary for G_1_/S cell cycle transition. Additionally, previous research has shown that PCNA-associated factor (*PCLAF)* acts as a regulator of DNA repair during DNA replication, and ubiquitin conjugating enzyme E2 C (*Ube2c)* is a protein-coding gene that encodes proteins necessary for the cell cycle to proceed (Hao, Zhang, & Cowell, 2012). Therefore, decreased mRNA expression levels of these genes after GaAD19 application in this study further confirmed that GaAD19 inhibited HeLa cell proliferation by blocking the cell cycle and inhibiting DNA synthesis.

The Western blot results showed that GaAD19 induced the activation of cleaved Caspase-9 and cleaved PARP1, which are involved in eukaryotic cell apoptosis, as well as *Bax*, a pro-apoptotic gene, while inhibiting the activity of the anti-apoptotic gene *Bcl-2*. Multiple pathways, including the MAPK, AMPK, and PI3K/Akt signaling pathways, are closely related to cell apoptosis. Based on the previous results, we next investigated the mechanism of action of GaAD19. Through bioinformatics analysis, we found that MAPK8 was one of its core targets. Molecular docking results showed that there was a strong binding energy between GaAD19 and JNK. At the same time, further experimental verification was carried out by Western blot. We detected other pathways in the MAPK pathway, such as p38 MAPK and ERK, and common cell proliferation regulatory pathways, such as AMPK and AKT, using Western blot. The experimental results showed that GaAD19 had no significant effect on these pathways. And we found that GaAD19 down-regulated the expression of JNK upstream activators MAP2K4 and MAP2K7 in a dose-dependent manner. G-coupled protein receptors play a key role in tumor progression and metastasis (Furukawa et al., 2022). Growth factors are multifunctional peptides involved in regulating cancer progression (Yaginuma et al., 2020). Cancer progression can cause significant cellular stress (Doolittle et al., 2022). qPCR results showed that GaAD19 regulated the JNK pathway by inhibiting the expression of growth factors, cellular stress and G_12/13_-coupled receptors, thereby inhibiting the proliferation and migration of cervical cancer cells. These experiments proved that JNK was the target of GaAD19.

To verify that GaAD19 mediates HeLa cell apoptosis through the JNK pathway, we performed quantitative analyses by counting the number of cell colonies after treating HeLa cells with SP600125, a JNK-specific inhibitor, and Anisomycin, a JNK-specific agonist. Administration of 5 μmol/L of GaAD19 along with 60 μmol/L SP600125 led to a decrease in the cell colony number as compared to those HeLa cells that were treated with 5 μmol/L GaAD19 alone. Contrastingly, the colony number of HeLa cells was significantly higher when treated with 5 μmol/L GaAD19 and 0.1 μmol/L Anisomycin compared to treatment with 5 μmol/L GaAD19 alone. These results indicated that the antiproliferative effect of GaAD19 on HeLa cells was reversed by the JNK agonist Anisomycin. After confirming that GaAD19 inhibited HeLa cell proliferation through the JNK signaling pathway, thus confirming the role of JNK pathway in HeLa cell apoptosis and cycle.

Through the establishment of a U14 cervical cancer xenograft model and trying different doses of GaAD19 in combination with JNK agonists to treat nude mice, we observed that GaAD19 can inhibit tumor growth in these mice by inhibiting the JNK pathway. Helper T cells (Th cells) play a very important role in the immune response. According to their biological functions, they can be divided into two different subsets: Th1 cell subsets secreting cytokines, such as IL-2 and TNF-*α*, and Th2 cell subsets secreting cytokines, such as IL-10 and IL-5 (Edwards et al., 2023). Under normal circumstances, Th1/Th2 is in a relatively balanced state, and the imbalance of Th1/Th2 will cause a disorder of the cytokine network, which will lead to the occurrence and development of many diseases. IL-2 can regulate the immune response of a variety of cells. It can cause antitumor effects, anti-microbial infections, anti-viral infections, and it can regulate the body’s immunity. IL-10 can inhibit the production of IL-2 by T cells and suppress the antitumor immune response, thus making the tumor further deteriorate (Arany, Grattendick, & Tyring, 2002). The results of this study showed that GaAD19 could significantly increase the content of IL-2 and decrease the content of IL-10 in serum from nude mice, while the effect of GaAD19 was weakened after being combined with JNK pathway agonists. This indicated that GaAD19 could enhance the antitumor immune response of the body by inhibiting the JNK pathway, thus inhibiting the further development of cervical cancer tumors. Although the antitumor effect of GaAD19 is weaker than that of CTX, it still has potential application prospects due to its low toxicity.

In conclusion, our results suggest that GaAD19 inhibits the JNK pathway and induces cervical cell apoptosis, thus realizing its anticancer effect on cervical cancer. Our findings provide robust evidence for the use of GaAD19 in the treatment of cervical cancer, and advocate further exploration of its usage in other medical conditions and diseases involving discrepant cell cycle division and cell growth.

## CRediT authorship contribution statement

**Mengchen Wang:** Writing – original draft, Validation, Methodology. **Qin Han:** Validation. **Xuelian Zhang:** Data curation. **Xi Dong:** Methodology. **Jiadong Ran:** Methodology, Writing – review & editing. **Fei Wei:** Conceptualization. **Yun Luo:** Writing – review & editing. **Xiaobo Sun:** Writing – review & editing, Funding acquisition.

## Declaration of Competing Interest

The authors declare that they have no known competing financial interests or personal relationships that could have appeared to influence the work reported in this paper.
