# Supplementary material for: Ganoderic acid a derivative induces apoptosis of cervical cancer cells by inhibiting JNK pathway
Source: Chin Herb Med. 2024 Jul 19;17(4):756–67. doi: 10.1016/j.chmed.2024.07.002 (PMC12702450; doi:10.1016/j.chmed.2024.07.002)
Supplement: Supplementary Data 1 [file mmc1.docx]

**Supplementary Material**


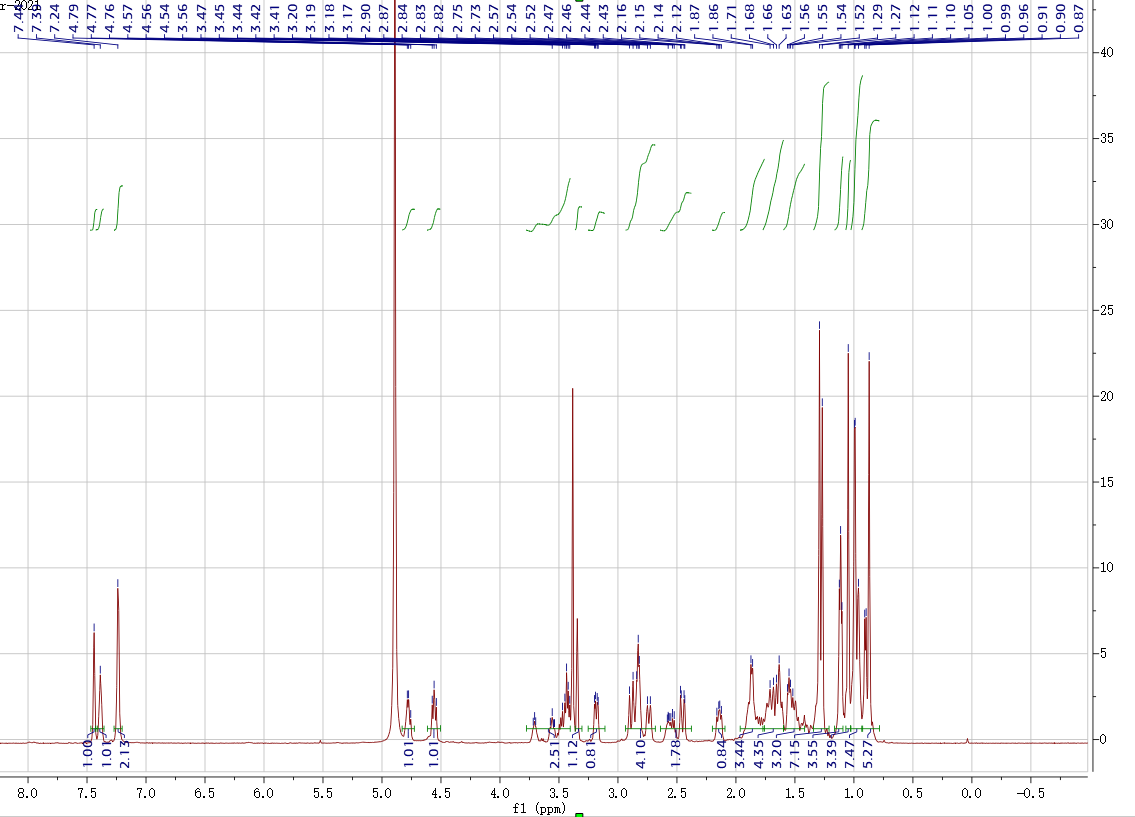


**Fig. S1.** ^1^H NMR of GaAD19.


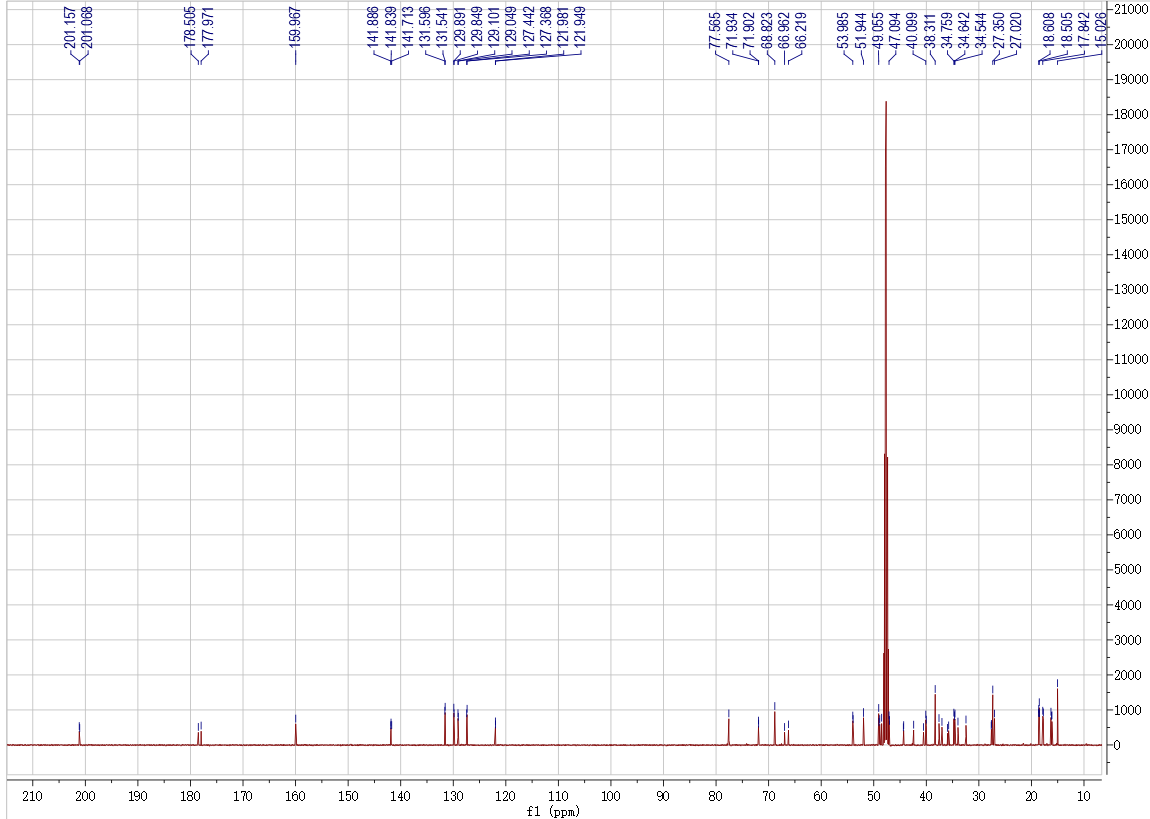


**Fig. S2.** ^13^C NMR of GaAD19.

**
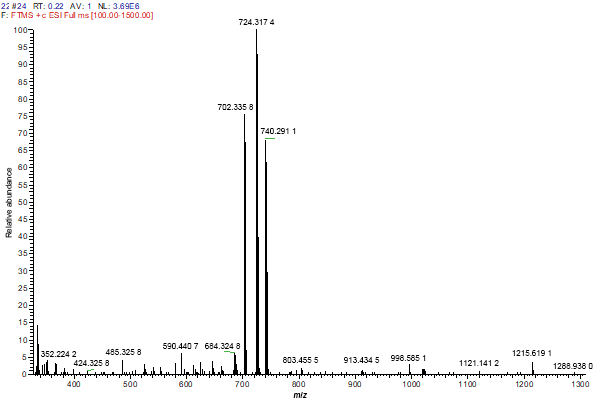
**

**Fig. S3.** HRMS of GaAD19.

**Table S1.** Cytotoxicity of GaAD19 against multiple cancer cell lines.

| Cells | IC_50_ (μmol/L) |
| --- | --- |
| HepG2 | 18.13 |
| MCF-7 | 21.55 |
| CAL27 | 17.22 |
| 231 | 33.62 |
| Lewis | 14.85 |
| Hela | 11.20 |
| H22 | > 100 |
| K562 | > 100 |
| HL60 | > 100 |
